# Introducing winter rice cropping by using non-saline tidal water influx in western basins of South 24 Parganas, India

**DOI:** 10.1038/s41598-020-80797-x

**Published:** 2021-01-12

**Authors:** Prasun Mukherjee, Subhasish Das, Asis Mazumdar

**Affiliations:** School of Water Resources Engineering, Jadavpur University, Kolkata, India

**Keywords:** Environmental sciences, Hydrology, Civil engineering

## Abstract

A population exceeding 3.8 million people in the western region of 24-Parganas (South) is directly or indirectly reliant on agriculture as their primary source of livelihood. The agricultural trend shows a clear lack of multi-cropping with a drop of nearly 30% in rice cultivation during the winter season. Nearly 50% of the region is directly dependent on canals. The introduction of tidal water in the canal network provides an exceptionally economical and highly effective mode of irrigation water supply. The primary aim of the study was to identify the cartographic characteristics and channel hydraulics in the summer season. It was noted that the canals have a wide discharge range of 0.03–540.03 m^3^/s, average evaporation loss of 9.07 mm/day with a seepage loss ranging from 0.04 to 6.36 m^3^/s. The tidal water ingress quantity was calculated to be 4.17 Mm^3^, 5.32 Mm^3^, 1.88 Mm^3^ at Diamond Harbour sluice (Sl.), Kulpi Sl. and Kholakhali Sl. respectively. It was denoted that the augmentation of tidal backwater six times monthly would suffice the winter crop water requirement for the majority of the basins. This would result in the production of 172.13 kt which was previously 17.6 kt resulting in an increase of production by 878.01%. The per capita income would also be increased by nearly 978% for the season, resulting in the macro-socioeconomic upliftment of the region.

## Introduction

The South 24 Parganas (S24 Pgns.) district of West Bengal, India has been found to have a record of an intense scarcity of rainfall (42 mm–11 mm–11 mm–24 mm) during the months November–December–January–February respectively^1^. This results in the need for irrigation water within the region for this extended period for effective agriculture and other relatable activities. Mono crop is the existing main crop pattern in the region due to these varied and seasonal rainfall activities throughout the year. Multi-cropping could enhance the productivity of the region. In the region, nearly 50% of the agricultural produce is directly dependent on canals. In the region, the tidal backwater is not only used for multi-cropping irrigation purposes and pisciculture but also to serve as a resource base for various other activities and opportunities like fodder production, brick manufacturing, and other microscale industries which directly and indirectly affect the socio-economic conditions of the occupants within the region. It also provides an effective way to utilize the excess freshwater which would otherwise be wasted into the Bay of Bengal if not managed properly. The global demand for water, energy, and food is increasing the stress on the resources which are utilized for supplying these basic commodities^2^. This system absolutely nullifies the requirement of any extra resources, further accentuating the supply of irrigation water and food supply proliferating the efficiency of the clean technology. Just a few studies give insight about different sorts of back-water inflow estimation done over the past decades. The backwater function was determined with the assistance of numerical integration technique^3^. The backwater surface profiles of rectangular channels at gradually varying flow conditions had also been described^4^. The estimation technique for the water level, when the stream is under steady change has also been introduced by using an expanded form of the Bernoulli's equation was applied^5^. A computational program has also been likewise discussed for composite backwater profiles in trapezoidal channels^6^. An arrangement for the augmentation of irrigation systems through an abundance of tidal back-water convergence for the region has been previously provided^7^.

The examination endeavored to depict not just the improvement of the financial return of the general population of the Magrahat basin yet additionally to make arrangements for a multi-cropping pattern of farming/cultivation including pisciculture options in the dry season for the sustenance of their employment. They further conjectured in light of the data and field studies that the Diamond Harbor Creek khal is an estuarine canal with a regular tidal influx. Particularly amid flash floods, the catchments used to be mostly flooded. To combat this issue, in the early 1960s, a strategy was set up by the Irrigation Department of the Government of West Bengal to regulate the passage of tidal ingress through a major sluice gate (Diamond Harbour sluice) and development of similar sluice gates in each estuarine canals (Kholakhali sluice, Hara sluice, Kulpi sluice) to check the surge issue in the territory as likewise to reserve the tidal backwater for use in lean periods. A proposal for the Magrahat basin irrigation management based on mathematical estimation had been examined^8^. The work was done purely on theoretical data collected from several sources and calculations were performed henceforth. Smaller reservoirs and branch canals are not considered here for the research as it would be very time consuming and difficult to reach certain sections physically. So, there might be a small difference (almost negligible) in the calculated results related to the water availability in the study area. The farmlands in the region do not usually directly receive water from the canals. There are several entrance points cut through the canal linings to receive water from the canals by the effect of tidal influx from the canals. It helps in a controlled yet ample water supply for cultivation. The water supply from the canals is then distributed via smaller channels or “nullah(s)” to the cultivation fields. The water is often even diverted to and stored in temporary storage ponds for future use when the canals do not receive enough water. The temporary storage ponds also are used for pisciculture, adding to the productivity of the reservoirs.

On several occasions whilst conducting the extensive field survey the people were noted to be curious and at several acquaintances enquired if the canals would be dredged or would be cleaned. It was uncanny how the population even being so dependent on the canal network was actively destroying its very backbone by dumping waste, sewage, and encroaching the very network that is so important for them. The lack of awareness and knowledge about the means to protect their asset is the most likely reason for such. The possibility to irrigate the total cultivatable command area of 523.18 km^2^ was examined with the sessional feeding of the tidal backwater for the period of water requirement i.e. from ground preparation to the last watering. The study would create provision for increasing the winter rice cultivation and also greatly supplement in enhancing the economic return of the population in the region.

## Study area

This study develops on a much broader area, covering five basins in the region the Magrahat basin, Keorapukur basin, Kholakhali basin, Hara-Hatuganj basin, and Kulpi basin (57 km^2^) of the S24 Pgns. play a major role in imparting freshwater via a system of integrated channel networks within the catchment for effective irrigation purposes. The Diamond Harbour sluice is one of the primary sources in the Magrahat basin that provides tidal backwater to the Diamond Harbour Creek which in turn provides water for the Dasani Khal, Nazra Khal, Upper Hatuganj Khal, Sangrampur Khal, Upper Dhanpota Khal, Joynagar Khal, Kata Khal, Usti Nainan Outfall Channel, Suryapur Inner Channel, Lower Keorapukur Khal, Hotor Khal, and Lower Srichanda Khal.

**Figure 1:**
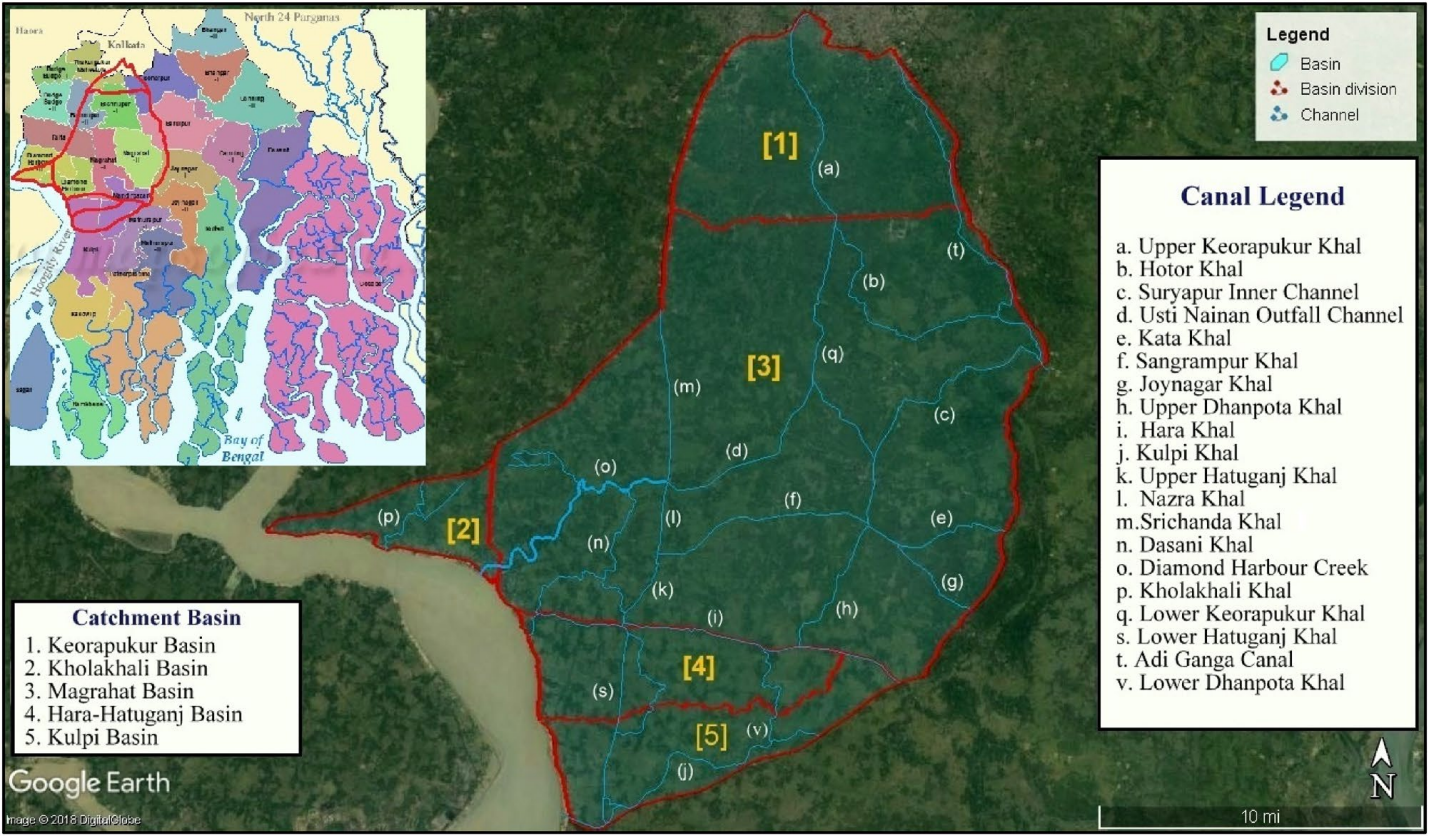
Study area.

The Kholakhali Khal which receives water from the Kholakhali sluice is the primary channel in the Kholakhali basin. The Hara Khal along with Mid Hatuganj Khal and Mid Dhanpota Khal receiving water from the Hara sluice plays the principal role in the tidal backwater irrigation process of Hara-Hatuganj basin. The Kulpi basin is primarily irrigated with water received from Kulpi sluice gate entering the Kulpi Khal, and a subsidiary water network formed by the Lower Hatuganj Khal and Lower Dhanpota Khal. Sustainable development is a subtle equilibrium between environmental protection, economic development, and societal upliftment which can be achieved only by efficient use of resources^9^. The study area was identified using Google earth software and the demarcations of the basins and canals in the study area were made for easy understanding of the region. The study area was subdivided into five basins that receive water directly from the Diamond Harbour sluice, Kholakhali sluice, Hara sluice, and the Kulpi sluice. The five basins in the S24 Pgns. where predominant tidal-backwater irrigation practices are being performed are mapped in Fig. 1.

## Methodology

A detailed survey was done to perceive the previous research work in literature like journals, conference proceedings, book chapters, and several statistical data reports. It was observed unfortunately that scanty work was carried out on the topic and on the study area. Government records on soil exploration and Land Use-Land Cover (LULC) maps of the study area were collected from Survey of India, Canals Division, and Magrahat drainage division of West Bengal Irrigation and Waterways Division. Field surveys of the region were carried out to verify the positioning of the canals with the maps collected. Satellite maps were extracted from United-States-Geological-Survey (USGS) Earth Explorer and the LULC map was prepared using ArcGIS v10.4 software and the canal paths were denoted on the satellite imagery as shown in Fig. 1. For a basic and clear understanding of the basin, the LULC map was prepared using ArcGIS v10.4 software. LANDSAT 8 OLI (Operational Land Imager) dataset was utilized having a 30 m spatial resolution of the year 2019. The accuracy of the LULC map was assessed using the Kappa coefficient. The overall accuracy and the Kappa coefficient value were 0.983 and 0.950, respectively. Later it was also validated with Google Earth imagery. The tidal timings and the tidal bore schedule were collected from Kolkata Port Trust Authorities, Ministry of Shipping, Government of India (GOI). Subsequent field visits were carried out for the collection of data (from November to February) to understand the characteristics of the channels during the tide and ebb effect. The cross-sectional (C/S) area of the channels was measured using echo-sounder, model GPSMAP 585, manufactured by Garmin, USA for before tide (BT) and after tide (AT) conditions. The readings were plotted in AutoCAD 2012 and the C/S area was calculated. Float method and piezometric depth variance measurement were carried out to find out the tidal velocity of the canals. The length of each canal was measured using the length scale conversion method on the corrected satellite imagery. The Area-velocity method was applied to find out the discharge of the canals at different points of testing. The actual discharge flowing through the canals differ from the calculated discharge as there is a prevalent conveyance loss in the canals. The quantity of available water can only be estimated after making deductions for conveyance loss. The conveyance loss was calculated as a summation of the seepage loss and evaporation loss as the operational loss could be considered negligible in this case. Irrigation systems are designed to perform at maximum efficiency conditions. It implies that the exchange of water at the base expense and with the least water loss^10^. The irrigated regions are extensive and henceforth require the transfer of water for irrigation purposes to travel longer distances. It leads to losses amid the transfer procedure. The canal lining is usually treated to limit such losses. However, the lining gradually deteriorates with the progression of time where cracks emerge that in the end lead to the collapse in the layers of lining. These cracks and fissures of covering joints are exit routes for water so necessary measures should be taken on time to diminish these losses^11^.

The Moritz equation (1967) was converted to a metric scale for this work. Seepage rates were estimated using Eq. (1) ^12^.

1 $$ S_{1} = 2366C\sqrt {{\raise0.7ex\hbox{$Q$} \!\mathord{\left/ {\vphantom {Q V}}\right.\kern-\nulldelimiterspace} \!\lower0.7ex\hbox{$V$}}} $$

where *S*_*1*_ is the seepage losses (m^3^.day^-1^.km^-1^); *Q* is the canal discharge (m^3^/s); *V* is the mean velocity of the canal (m/s); *C* is the coefficient directly varying on the function of the material (soil type) used to construct the canal lining. The modified A. N. Kostiakov equation (1978) was also used for re-estimating the seepage losses from earthen canals (Eq. 2) ^13^.

2 $$ S_{2} = s\frac{{QL_{1} }}{100} $$

where; *S*_2_ is the seepage losses (m^3^/s); *L*_1_ is the length of the canal (km); *s* is the water losses per km of canal length (%) was calculated using Eq. (3), where *A* and *m* are empirical constants depending on soil permeability.

3 $$ s = \frac{A}{{Q^{m} }} $$

Soil samples were collected from the canals for testing the soil type of the canals. The grading of the soil samples was performed by the sieve analysis method. Soil samples collected from the canals were tested using the variable head permeability test to determine the permeability of the underlying soil. Variable head permeameter was primarily used to measure the permeability of relatively less pervious soil. The coefficient of permeability, *K* (cm/s) was calculated using Eq. (4), where *h*_1_ is the initial head (cm); *h*_2_ is the final head (cm); *t* is the time interval (min); *a* is the C/S area of the standpipe (cm^2^); *A* is the C/S area of the specimen (cm^2^); *L*_2_ is the length of specimen.

4 $$ K = \frac{{2.3aL_{2} }}{At}\log_{10} \frac{{h_{1} }}{{h_{2} }} $$

Apart from seepage loss, the canals are also subjected to evaporation loss. Evaporation loss is dependent on several climatological parameters. Considering the several climatological parameters the evaporation loss has been calculated using Meyer’s equation (5).

5 $$ E_{L} = K_{M} \left( {e_{w} - e_{a} } \right)\left( {1 + \frac{{u_{9} }}{16}} \right) $$

where *E*_*L*_ is the evaporation (mm/day); *K*_*M*_ is the coefficient with a value of 0.36 for large deep waters and 0.50 for small, shallow waters; *e*_*w*_ is the saturated vapor pressure at the water surface (mm of mercury); *e*_*a*_ is the actual vapor pressure of overlying air at specified height (mm of mercury); *H*_*r*_ is the relative humidity (Eq. 6); *u*_*9*_ is the monthly mean wind velocity at 9 m above ground level (GL) (km/h)

$$ e_{a} = \, H_{r} \, \times \, e_{w} $$

In this scenario, the wind velocity was collected from the Indian Meteorological Department (IMD), GOI reports, which were collected at a height of 1 m above ground level, so it had to be converted to find the wind velocity at 9 m above GL. The wind velocity was calculated using the 1/7 power law (Eq. 7).

7 $$ u_{9} = \, u_{1} 9^{\frac{1}{7}} $$

where and *u*_*1*_ is the wind velocity at 1 m above GL (km/h).

**Figure 2:**
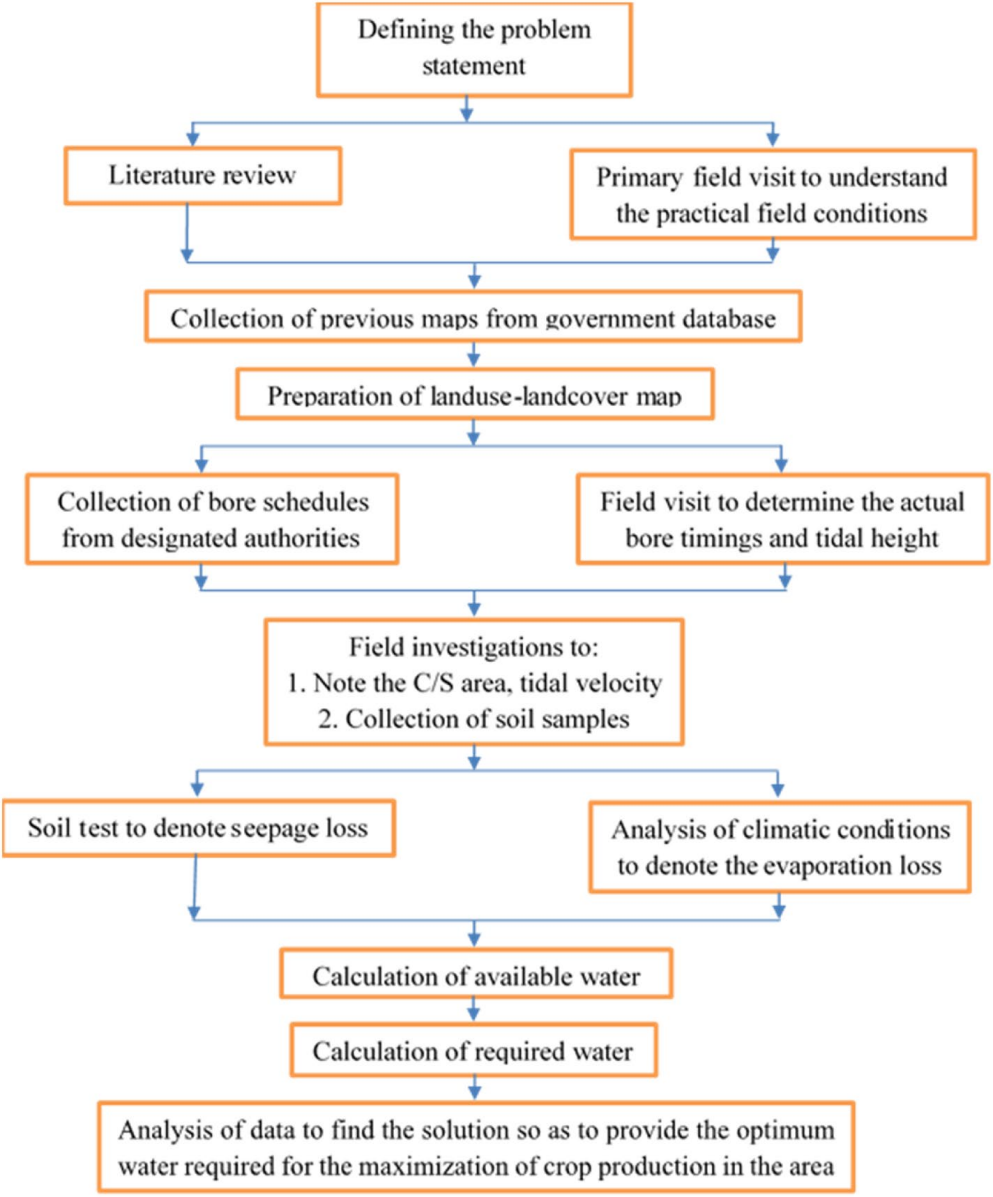
Simplified flowchart of methodology.

The total available water volumes at ebb and tide conditions were calculated from field data. The total basin area, gross cultivable area (GCA), and cultivable command area (CCA) were calculated from the LULC data. The climatic and meteorological data were extracted from CLIMWAT 2.0 software for Sagar Island recording station as it was the nearest to the climatic conditions of the region. The data were then fed into the CROPWAT 8.0 software. The CROPWAT software uses rainfall data to indicate the effective rainfall for the growth of crops in the region. The effective rainfall was calculated in the software by the United States Department of Agriculture Soil Conservation Service Method (USDA-SCS). Effective rainfall is required as not all the quantity of rainfall that the catchment area receives can be used due to surface runoff and deep percolation below the root zone. Other parameters as the max–min temperature, wind speed, relative humidity, and average sun hours were also provided for the region and were also input into the software for consideration. The crop characteristics were taken from the CROPWAT database. The two primary factors which CROPWAT soil template considers are the available water storage capacity of the soil and the daily final capacity of rainfall infiltration. The available water storage capacity is the same for both black soil and clayey loam soil i.e. 200 mm/m^14^. The daily final capacity of rainfall infiltration for black soil is 30 mm/day and for clayey loam is 30.48 mm/day, which can also be considered as identical^15^. Another secondary and notable consideration in the template is the maximum root depth. The template has an allowance for 900 cm whilst the maximum root depth for rice ranges from 55 to 65 cm, so abundant allowance has been considered on using the template^16,17^. Henceforth, the black clay soil template was used in this part of the research. Now, from the water availability and crop requirements, the deficiency was calculated to note the ideal number of times the sluice gates need to open to allow the tidal influx to occur in the region to increase the production rate of Winter Rice in the region. It is to be noted that the available water was calculated from the water entering the canal network from Hooghly River at the definitive sluice gates that are opened to allow the water to enter during the high tide and is closed after the peak period of the tide. The period of the high tide ranges from a period of 4.5–5.5 h so an average of 5 h has been considered as the mean to calculate the average tidal water influx into the basins. The tidal water entering was then reproduced by two, four, and six times to calculate the average monthly water availability for a specific month and basin to find out the water availability in the event of tidal water ingress. Propositions have been made according to the results for multi-cropping according to net-water availability. A concise flowchart of the methodology has been shown in Fig. 2.

## Results

**Figure 3:**
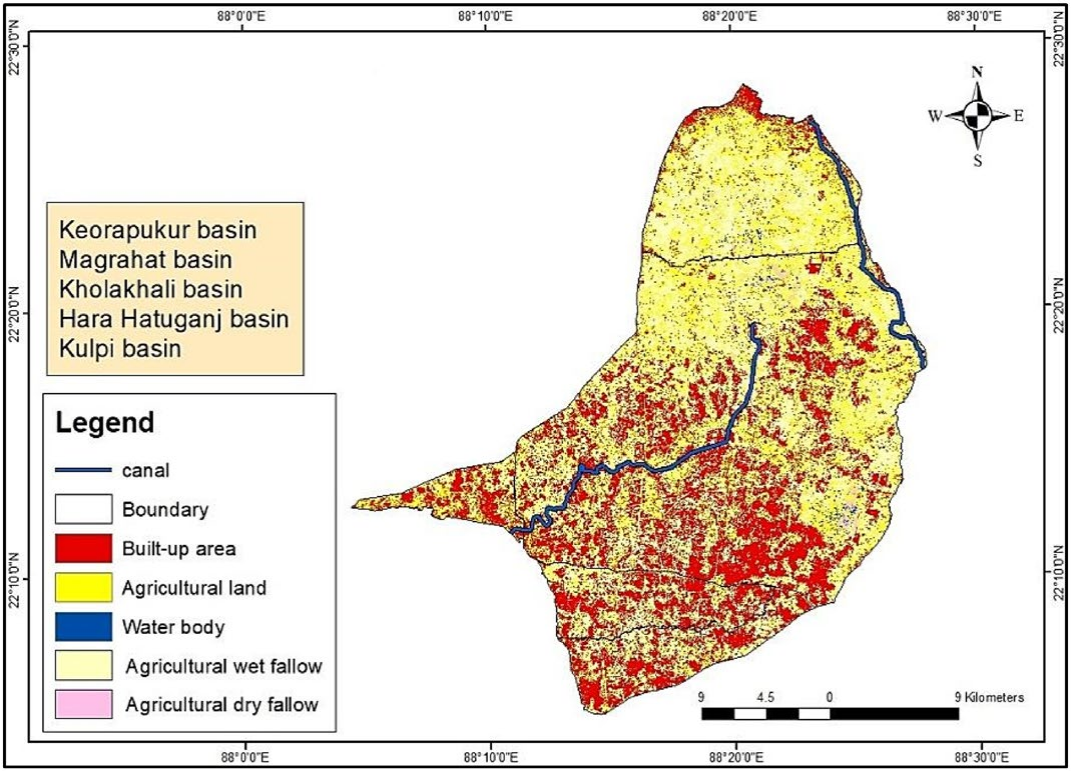
LULC map of study area, prepared in ArcGIS v10.4.

The LULC map of the region showed a difference in data from actual field data. Henceforth, ground-truthing was done as an additional reference and to prepare the adjusted LULC map, and data is depicted in Fig. 3 and Table 1 respectively. The field-collected data for canal length, the cross-sectional area before tide (C/S area BT), the cross-sectional area after tide (C/S area AT) and calculated discharge has been shown in Table 2.

**Table 1:** LULC distribution.

Basin name		Built-up area	Agricultural cropland	Water-body	Agricultural dry land	Wet-land	Total area	CCA
Kulpi basin	Area (km^2^)	28.85	6.81	0.05	22.09	0.02	57.82	28.91
	% to area of the basin	49.90	11.80	0.10	38.20	~ 0.00		49.90
Hara-Hatuganj basin	Area (km^2^)	28.67	6.28	0.03	23.32	0.01	58.32	29.61
	% to area of the basin	49.20	10.80	0.10	40.0	~ 0.00		50.80
Kholakhali basin	Area (km^2^)	13.61	9.37	0.09	6.41	0.03	29.51	15.78
	% to area of the basin	46.10	31.70	0.30	21.70	0.10		53.50
Magrahat basin	Area (km^2^)	170.65	118.75	3.24	216.14	5.28	514.06	334.89
	% to area of the basin	33.20	23.10	0.60	42.0	1.00		65.10
Keorapukur basin	Area (km^2^)	16.83	47.39	1.68	66.61	1.87	134.38	114.0
	% to area of the basin	12.50	35.30	1.20	49.60	1.40		84.80

*CCA* cultivable command area.

**Table 2:** Field collected data and calculated discharge.

Canal name	Length (km)	Canal code	Pt No	C/S area BT (m^2^)	C/S area AT (m^2^)	Mean C/S area (m^2^)	% incr. AT	Tidal vel. (m/s)	Calculated discharge (m^3^/s)
Upper Keorapukur Khal	14.07	A	1	3.73	3.73	3.73	0	UD#	0
			2	5.24	7.94	6.59	51.50	0.42	2.76
Hotor Khal	19.68	B	1	63.38	129.45	96.41	104.20	0.48	46.28
			2	50.50	108.31	79.41	114.50	0.84	66.70
			3	19.05	27.28	23.17	43.10	1.02	23.63
			4	8.09	16.43	12.26	103.10	1.10	13.49
Suryapur Inner Channel	16.61	C	1	48.94	106.97	77.95	118.60	1.71	133.31
			2	40.69	54.85	47.77	34.80	1.56	74.53
			3	37.19	48.85	43.02	31.40	1.62	69.70
			4	33.94	44.27	39.11	30.40	1.70	66.48
Usti Nainan Outfall Channel	10.8	D	1	47.64	105.57	76.61	121.60	1.20	91.93
			2	182.74	306.00	244.37	67.50	1.40	342.12
Kata Khal	7.97	E	1	39.37	51.71	45.54	31.40	0.80	36.43
			2	33.94	44.97	39.46	32.50	1.90	74.96
Sangrampur Khal	11.78	F	1	38.59	50.94	44.76	32.00	0.50	22.38
			2	43.51	61.02	52.26	40.20	1.12	58.54
Joynagar Khal	5.30	G	1	39.75	53.07	46.41	33.50	1.67	77.51
			2	2.08	3.84	2.96	84.20	1.64	4.86
Upper Dhanpota Khal	7.88	H	1	20.02	27.63	23.83	38.00	0.3	7.15
			2	14.04	21.06	17.55	50.00	0.12	2.11
Hara Khal	21.09	I	1	74.29	123.07	98.68	65.70	2.42	238.82
			2	16.49	25.48	20.99	54.50	1.79	37.57
			3	17.25	26.74	21.99	55.00	1.23	27.05
			4	5.01	8.77	6.89	74.90	0.72	4.96
			5	39.39	39.39	39.39	0.00	0.48	18.91
Kulpi Khal	16.40	J	1	50.65	98.83	74.74	95.10	2.36	176.40
			2	21.33	40.55	30.94	90.10	2.01	62.19
			3	21.33	40.55	30.94	90.10	1.74	53.84
			4	11.63	17.60	14.62	51.30	1.08	15.79
			5	11.63	17.60	14.62	51.30	0.82	11.98
Upper Hatuganj Khal	3.75	K	1	5.92	9.98	7.95	68.50	0.53	4.21
			2	6.01	10.06	8.04	67.50	0.34	2.73
Nazra Khal	3.73	L	1	13.31	19.56	16.43	46.90	0.63	10.35
			2	N/A	N/A	N/A	N/A	N/A	N/A
Srichanda Khal	9.29	M	1	67.68	114.24	90.96	68.80	0.75	68.22
			2	0.71	3.46	2.09	382.30	UD#	0.00
Dasani Khal	16.19	N	1	13.17	19.42	16.29	47.40	1.34	21.84
			2	12.17	14.45	13.31	18.80	0.66	8.78
			3	37.38	55.84	46.61	49.40	1.42	66.19
			4	4.07	4.07	4.07	0.00	0.53	2.16
Diamond Harbour Creek	15.33	O	1	186.12	309.73	247.93	66.40	2.40	595.02
			2	187.46	305.55	246.51	63.00	2.02	497.95
Kholakhali Khal	6.63	P	1	68.54	121.06	94.80	76.60	1.99	188.65
			2	11.73	32.03	21.88	173.00	0.72	15.76
			3	N/A	N/A	N/A	N/A	N/A	N/A
Lower Keorapukur Khal	14.68	Q	1	15.13	22.89	19.01	51.30	1.30	24.71
			2	29.98	42.89	36.44	43.00	1.20	43.73
			3	5.63	11.19	8.41	98.70	1.20	10.09
			4	19.27	23.65	21.46	22.70	1.60	34.34
Lower Hatuganj Khal	9.98	S	1	6.11	10.25	8.18	67.80	0.82	6.71
			2	14.15	21.33	17.74	50.80	0.32	5.67
Adi Ganga Canal	17.81	T	1	2.36	7.30	4.83	208.40	0.63	3.05
			2	44.20	57.71	50.95	30.50	0.91	46.37
Lower Dhanpota Khal	6.27	V	1	0.34	0.82	0.58	138.80	0.24	0.14
			2	1.38	1.92	1.65	39.30	UD#	0

*N/A* not applicable, *UD#* undetectable, *C/S* cross-sectional, *incr.* increase, *AT* after tide, *BT* before tide.

It was noticed during the field survey that the canals were unlined non-prismatic channels. The results as depicted in Table 3 show that the canal substrate is primarily clayey or loamy in nature. Moritz suggested the value of the co-efficient for clay and clayey loam as 0.41. Henceforth, following Eq. (1), the seepage value of the canals was calculated and the results are depicted in Table 3.

**Table 3:** Soil type and canal seepage according to Moritz's equation.

Point name	Soil type	Mean velocity (m/s)	Mean discharge (m^3^/s)	Seepage per km (m^3^ day^−1^ km^−1^)	Canal seepage (m^3^/s)
Upper Keorapukur Khal	Clayey	0.21	1.33	3437.94	0.58
Hotor Khal	Clayey	0.86	37.52	8845.63	2.08
Suryapur Inner Channel	Loamy	1.64	86	9675.04	1.92
Usti Nainan Outfall Channel	Loam	1.3	217.03	17,301.7	2.24
Kata Khal	Loamy	1.35	55.7	8601.28	0.82
Sangrampur Khal	Clayey Loam	0.81	40.46	9464.21	1.33
Joynagar Khal	Loamy	1.65	41.18	6680.07	0.42
Upper Dhanpota Khal	Loamy	0.21	4.62	6286.14	0.59
Hara Khal	Clayey	1.32	65.46	9401.67	2.38
Kulpi Khal	Clayey	1.6	64.04	8466.56	1.66
Upper Hatuganj Khal	Loamy	0.43	3.47	3785.09	0.17
Nazra Khal	Loamy	0.32	10.35	7677.49	0.34
Srichanda Khal	Clayey	0.37	34.11	12,771.2	1.42
Dasani Khal	Clayey	0.98	24.74	6703.04	1.3
Diamond Harbour Creek	Loamy	2.21	546.48	21,056.9	3.87
Kholakhali Khal	Clayey	1.35	102.21	11,629.8	0.93
Lower Keorapukur Khal	Loamy	1.32	28.22	6179.88	1.09
Lower Hatuganj Khal	Loamy	0.57	6.19	4414.16	0.53
Adi Ganga Canal	Clayey Loam	0.77	24.71	7585.59	1.62
Lower Dhanpota Khal	Clayey	0.12	0.07	1021.95	0.08

The values of *h*_*1*_, *h*_*2*_, *t* are variables for every soil type and the tested results and calculations are shown in Table 4. The values of *a* (0.196 cm), *A* (78.53 cm), and *L* (13 cm) are constant for all the calculations, in this case. The permeability (*P*) of the canal linings shows that the canals have a low permeability rate. Henceforth, the value of constants ‘*A*’ is 0.7 and ‘*m*’ is 0.3. The seepage rates of each canal according to modified Kostiakov’s equation have been depicted in Table 4. The seepage values retrieved using the Moritz equation and modified Kostiakov’s equation although quite near but the values differ to some extent. Henceforth, for the calculation of losses, the average of the values was taken into account. This would provide a singular value, reducing confusion and ease further calculations. The mean seepage value has been calculated in Table 5.

**Table 4:** Seepage of the canals according to modified Kostiakov's equation.

Canal name	*t* (min)	*h*_*1*_ (cm)	*h*_*2*_ (cm)	*K* (cm/s)	P	*A*	*m*	*s* (%)	Seepage of canal (m^3^/s)
Upper Keorapukur Khal	250	40	35	4.15 × 10^–7^	Low	0.7	0.3	0.63	0.12
Hotor Khal	600	40	36	9.48 × 10^–8^	Low	0.7	0.3	0.23	1.74
Suryapur Inner Channel	18	50	33	1.24 × 10^–5^	Low	0.7	0.3	0.18	2.62
Usti Nainan Outfall Channel	90	50	38	1.64 × 10^–6^	Low	0.7	0.3	0.14	3.26
Kata Khal	48	50	32	5.02 × 10^–6^	Low	0.7	0.3	0.21	0.93
Sangrampur Khal	80	50	42	1.17 × 10^–6^	Low	0.7	0.3	0.23	1.09
Joynagar Khal	22	50	28	1.42 × 10^–5^	Low	0.7	0.3	0.22	0.5
Upper Dhanpota Khal	75	40	25	3.38 × 10^–6^	Low	0.7	0.3	0.44	0.16
Hara Khal	105	50	42	8.96 × 10^–7^	Low	0.7	0.3	0.19	2.75
Kulpi Khal	130	40	36	4.37 × 10^–7^	Low	0.7	0.3	0.20	2.11
Upper Hatuganj Khal	90	40	28	2.14 × 10^–6^	Low	0.7	0.3	0.48	0.06
Nazra Khal	15	40	32	8.03 × 10^–6^	Low	0.7	0.3	0.34	0.13
Srichanda Khal	178	40	34	4.93 × 10^–7^	Low	0.7	0.3	0.24	0.76
Dasani Khal	175	50	38	8.47 × 10^–7^	Low	0.7	0.3	0.26	1.07
Diamond Harbour Creek	25	40	25	1.01 × 10^–5^	Low	0.7	0.3	0.10	8.85
Kholakhali Khal	95	40	35	7.59 × 10^–7^	Low	0.7	0.3	0.17	1.18
Lower Keorapukur Khal	17	40	28	1.13 × 10^–5^	Low	0.7	0.3	0.25	1.06
Lower Hatuganj Khal	30	40	22	1.07 × 10^–5^	Low	0.7	0.3	0.41	0.25
Adi Ganga Canal	106	40	30	1.46 × 10^–6^	Low	0.7	0.3	0.26	1.17
Lower Dhanpota Khal	145	40	34	3.38 × 10^–6^	Low	0.7	0.3	1.55	0.01

*K* coefficient of permeability, *h*_*1*_ initial head, *h*_*2*_ final head, *t* time interval, *P* permeability, *A,m* permeability constants, *S%* water losses per km of canal length.

**Table 5:** Mean seepage loss.

Canal name	Total seepage of canals (m^3^/s)		Mean seepage (m^3^/s)
	Moritz (1967)	Modified Kostiakov (1978)	
Upper Keorapukur Khal	0.58	0.12	0.35
Hotor Khal	2.09	1.74	1.91
Suryapur Inner Channel	1.93	2.62	2.27
Usti Nainan Outfall Channel	2.24	3.26	2.75
Kata Khal	0.82	0.93	0.87
Sangrampur Khal	1.34	1.10	1.22
Joynagar Khal	0.42	0.50	0.46
Upper Dhanpota Khal	0.59	0.16	0.37
Hara Khal	2.37	2.75	2.56
Kulpi Khal	1.66	2.11	1.88
Upper Hatuganj Khal	0.17	0.06	0.12
Nazra Khal	0.34	0.13	0.23
Srichanda Khal	1.42	0.77	1.09
Dasani Khal	1.30	1.07	1.19
Diamond Harbour Creek	3.87	8.84	6.36
Kholakhali Khal	0.92	1.18	1.05
Lower Keorapukur Khal	1.08	1.06	1.07
Lower Hatuganj Khal	0.52	0.25	0.38
Adi Ganga Canal	1.62	1.17	1.39
Lower Dhanpota Khal	0.07	0.01	0.04

Tables 6 and 7 show the canal surface area with temperature and the relative humidity with wind velocity respectively. Now according to Eqs. (6), (7), and (5) the values *e*_*a*_ and *u*_9_ and E_L_ were calculated to be 7.02 mm of Hg, 11.58 kmph, and 9.07 mm/day respectively. The mean seepage loss and the evaporation loss were deducted from the mean discharge to calculate the actual discharge for the canals, as shown in Table 8. The discharge of the canals is to be noted is for the opening of the sluice gate at the event of only one tide as the canals have virtually no other source of water entering in addition to the tidal waters of Hooghly River.

**Table 6:** Canal surface area and temperature.

Canal name	Average top width (m)	Surface area (m^2^)	Avg. water temp. (°C)	Avg. water temp. (°C)	*e*_*w*_
Upper Keorapukur Khal	12.25	172,357.50	17.25	19.931 ≈ 20	17.54
Hotor Khal	42.55	837,507.00	21.70		
Suryapur Inner Channel	29.35	487,595.14	21.50		
Usti Nainan Outfall Channel	53.68	579,663.64	19.75		
Kata Khal	23.50	187,154.00	20.75		
Sangrampur Khal	29.99	353,221.67	20.50		
Joynagar Khal	17.93	95,011.07	21.75		
Upper Dhanpota Khal	14.61	115,136.32	20.75		
Hara Khal	18.56	391,575.51	3.54		
Kulpi Khal	25.74	422,126.66	20.62		
Upper Hatuganj Khal	13.56	50,863.56	20.25		
Nazra Khal	15.59	58,181.88	18.50		
Srichanda Khal	19.99	185,793.54	22.05		
Dasani Khal	16.17	261,905.60	20.67		
Diamond Harbour Creek	60.79	931,926.56	20.25		
Kholakhali Khal	20.79	137,816.91	20.50		
Lower Keorapukur Khal	22.08	324,222.72	20.62		
Lower Hatuganj Khal	14.09	140,685.43	21.55		
Adi Ganga Canal	25.87	460,685.74	23.75		
Lower Dhanpota Khal	5.19	32,577.93	22.35		

*Avg.* average, *e*_*w*_ saturated vapor pressure at the water surface (mm of mercury).

**Table 7:** Relative humidity and wind velocity of the study area.

Year	Nov		Dec		Jan		Feb		Annual (*H*_*r*_ %)	Annual (*u*_*1*_)	Decennial (*H*_*r*_ %)	Decennial (*u*_*1*_)
	*H*_*r*_	*u*_*1*_	*H*_*r*_	*u*_*1*_	*H*_*r*_	*u*_*1*_	*H*_*r*_	*u*_*1*_				
2009	66	5.8	49	5.8					57.50	5.80	50.68	8.46
2010	43	6.3	51	8.7	67	7.6	60	6.7	55.25	7.30		
2011	62	7.0	51	8.8	53	8.3	53	8.7	54.75	8.20		
2012	58	10.4	45	10.5	56	8.8	48	8.6	51.75	9.50		
2013	65	10.0	52	9.4	43	9.3	40	9.7	50.00	9.60		
2014	63	8.5	41	10	45	10.2	52	8.9	50.25	9.40		
2015	56	6.7	49	7.5	48	9.6	49	9.1	50.50	8.20		
2016	58	7.2	45	8.0	48	6.8	58	7.2	52.25	7.30		
2017	57	9.1	50	8.8	39	8.3	47	7.1	48.25	8.30		
2018	56	8.1	43	11.4	35	8.8	44	8.8	44.50	9.20		
2019					38	9.7	47	10.4	42.50	10.10		

*H*_*r*_ relative humidity, *u*_*1*_ wind velocity at 1 m above ground level.

**Table 8:** Actual discharge of the canals.

Canal name	Mean discharge before loss deduction (m^3^/s)	Canal seepage loss (m^3^/s)	Canal evaporation loss (m^3^/s)	Canal actual discharge (m^3^/s)
Upper Keorapukur Khal	1.38	0.35	18.088 × 10^–3^	1.01
Hotor Khal	37.52	1.91	87.893 × 10^–3^	35.52
Suryapur Inner Channel	86.00	2.27	51.171 × 10^–3^	83.67
Usti Nainan Outfall Channel	217.03	2.75	60.833 × 10^–3^	214.21
Kata Khal	55.70	0.87	19.641 × 10^–3^	54.80
Sangrampur Khal	40.46	1.21	37.069 × 10^–3^	39.2
Joynagar Khal	41.18	0.46	9.971 × 10^–3^	40.71
Upper Dhanpota Khal	4.62	0.37	12.083 × 10^–3^	4.23
Hara Khal	65.46	2.56	41.094 × 10^–3^	62.85
Kulpi Khal	64.04	1.88	44.300 × 10^–3^	62.11
Upper Hatuganj Khal	3.47	0.11	5.338 × 10^–3^	3.35
Nazra Khal	10.35	0.23	6.106 × 10^–3^	10.11
Srichanda Khal	34.11	1.10	19.498 × 10^–3^	32.99
Dasani Khal	24.74	1.18	27.486 × 10^–3^	23.53
Diamond Harbour Creek	546.48	6.36	97.802 × 10^–3^	540.03
Kholakhali Khal	102.21	1.05	14.463 × 10^–3^	101.13
Lower Keorapukur Khal	28.22	1.07	34.026 × 10^–3^	27.11
Lower Hatuganj Khal	6.19	0.38	14.764 × 10^–3^	5.79
Adi Ganga Canal	24.71	1.39	48.347 × 10^–3^	23.26
Lower Dhanpota Khal	0.06	0.04	3.419 × 10^–3^	0.03

Since the research concentrates on maximizing the production of the winter rice in the region, the CCA was considered as the total area where rice is currently cultivated. The observed trend shows that whilst for monsoon rice 38.27% of the CCA is used but for winter rice only 8.249% of the CCA is used. This is a drop of nearly 30% in the growing of rice in the winter season.

As mentioned earlier, the canals receive the bulk of their water from tidal ingress. Hara-Hatuganj basin and Kulpi basin were considered co-jointly as it was noted during the field visits and also in satellite imagery that the canal network for the two basins was so intensely interwoven that differentiating the basins would provide an error as the likelihood of water distributing itself easily is very high. As the tidal velocity of the points were previously measured in the field visits. The tidal discharge was calculated as shown in Table 9. The discharge into the Keorapukur basin is from the water entering from the Magrahat basin and is thus lost to the Magrahat basin. So, the water entering the Keorapukur basin has been deducted from the water calculated to be available for the Magrahat basin. The high tide is noticed to last an average of 5 h per event, for an average of two events per month. The total monthly availability was calculated henceforth as depicted in Table 10.

**Table 9:** Water availability due to one tidal influx.

Canal name	Hara Khal	Kulpi Khal	Diamond Harbour Creek	Kholakhali Khal	Upper Keorapukur Khal
C/S area before tide (m^2^)	74.29	50.66	186.13	68.54	5.24
C/S area after tide (m^2^)	123.08	98.84	309.73	121.06	7.94
Velocity (m/s)	2.42	2.36	2.40	1.99	0.42
Tidal discharge in the canals (m^3^/s)	118.06	113.70	296.65	104.52	1.13
Quantity of tidal water entering (Mm^3^)	2.13	2.05	5.35	1.88	0.02
Quantity of tidal water usable for irrigation in one tide per basin (Mm^3^)	4.17		5.32	1.88	0.02

**Table 10:** Monthly water requirement for winter rice.

	Oct	Nov	Dec	Jan	Feb	Mar
Crop water requirement (m/month)	0.09	0.20	0.09	0.09	0.09	0.07
Keorapukur basin (Mm^3^)	10.31	23.22	10.61	9.92	10.18	7.68
Magrahat basin(Mm^3^)	30.27	68.22	31.18	29.14	29.91	22.57
Kholakhali basin (Mm^3^)	1.43	3.21	1.47	1.37	1.41	1.06
Hara-Hatuganj & Kulpi basin (Mm^3^)	5.29	11.92	5.45	5.09	5.23	3.94

*Oct* october, *Nov* november, *Dec* december, *Jan* january, *Feb* february, *Mar* march.

This was then deducted from the average monthly water requirement of the crops which had been calculated earlier in Table 10 to find out the deficiency (if any) and the deficiency percentage (if any) for the basins for that particular month. It was also noted to take into account that the efficiency of the system remains high, i.e. the number of times the gates need to be opened be kept minimum so as to also reduce the operational cost. Furthermore, excess water entering the canal network could also result in potential flooding of the area of the basins adjoining the riverbank, as was noted earlier. The details of the calculations are shown in Table 11. The results show that the Keorapukur basin has a deficiency of more than 98% even after the tidal water influx happening more than six times, which is a major lag for the region. It is suggested that the basin opts for other means of crop cultivation. It is to be also noted that the recent advancements of the socio-economic and geo-political trend of the region are pointing towards a drastic change in the livelihood of the people. The beneficiary (i.e. inhabitants of the region) are changing to a more suburban kind of livelihood due to recent progress of the Kolkata city establishments distributing further in those regions. The shifting of several government administrative units from the central region of the Kolkata city to the regions and adjoining indicate a massive turn in the dependence of livelihood of the people in the region. The trends suggest that the ever-expanding Kolkata city is most likely to grasp the region into an urban-suburban kind of region where agriculture will not be a major key player, and the canal network might serve only as a part of an efficient drainage system. For the remaining other basins after careful analysis of the calculated data, the following plan of action is being suggested so as to get the maximum productivity in Table 12.

**Table 11:** Calculations for feeding backwater tidal influx.

		Keorapukur basin			Magrahat basin			Kholakhali basin	Hara-Hatuganj & Kulpi basins	
Proposed no. of feedings		2	4	6	2	4	6	2	2	4
Mean monthly water availability (Mm^3^)		0.04	0.08	0.12	10.64	21.27	31.91	3.76	10.64	16.68
Oct	Monthly deficiency (Mm^3^)	10.26	10.22	10.18	19.63	8.99	− 1.64	− 2.336	− 3.05	− 11.39
	Deficiency (%)	99.61	99.21	98.81	64.86	29.72	N/A	N/A	N/A	N/A
Nov	Monthly deficiency (Mm^3^)	23.18	23.14	23.1	57.58	46.93	36.3	− 0.54	3.57	− 4.76
	Deficiency (%)	99.83	99.65	99.47	84.41	68.81	53.21	N/A	30	N/A
Dec	Monthly deficiency (Mm^3^)	10.57	10.53	10.49	20.53	9.9	− 0.74	− 2.29	− 2.89	− 11.23
	Deficiency (%)	99.62	99.23	98.85	65.88	31.76	N/A	N/A	N/A	N/A
Jan	Monthly deficiency (Mm^3^)	9.87	9.83	9.79	18.49	7.85	− 2.78	− 2.38	− 3.25	− 11.59
	Deficiency (%)	99.59	99.19	98.77	63.49	26.97	N/A	N/A	N/A	N/A
Feb	Monthly deficiency (Mm^3^)	10.13	10.09	10.06	19.26	8.62	− 2.01	− 2.35	− 3.11	− 11.46
	Deficiency (%)	99.6	99.19	98.79	64.43	28.85	N/A	N/A	N/A	N/A
Mar	Monthly deficiency (Mm^3^)	7.64	7.6	7.56	11.93	1.29	− 9.35	− 2.69	− 4.39	− 12.74
	Deficiency (%)	99.47	98.94	98.41	52.87	5.73	N/A	N/A	N/A	N/A

**Table 12:** Schedule for the sluice gates to allow tidal water influx.

	DH sluice	Kholakhali sluice	Hara sluice	Kulpi sluice
Month	Number of times the gates need to be opened			
October	6	1	2	2
November	6	2	4	4
December	6	1	2	2
January	6	1	2	2
February	6	1	2	2
March	6	1	2	2

*DH* diamond harbour.

It is to be noted that even after allowing tidal water influx six times in November at the Diamond Harbour sluice there was a deficiency of 53.21% or 36.3 m^3^ winter irrigation water. This could be easily avoided by channeling water from other basins in the network to enter the basin and also by supplementing water from the Piyali River in the east of the basin during November to fulfill that need. It was noted from the population data recorded in Census 2011 that the rural population has an average family with one earning member per family has a size of 5–12 members. The proper irrigation management of the study area would directly benefit nearly 3.8 million people. Since the current yield rate of winter rice in the region is 3290 kg/ha, the inclusion of the plan would result in the production of 172.13 kt which was previously only 17.6 kt. The increased area of cultivation could likewise provide an opportunity to employ more than 4.6 million new farmers, which will greatly supplement the agricultural sector not just within the region but also the whole nation. The average income of a farmer per harvest was nearly INR 892, which greatly dissuaded farmers in winter rice harvesting. The current plan could increase the income to INR 8724 per harvest per farmer, which is nearly ten times or a 978% uptrend.

## Discussions

Satellite imagery models are helpful tools to comprehend the conduct of hydrological systems. Quite unfortunately, the parameterization of these hydrological models requires a wide range of information, from various sources, and from various orders (e.g., meteorological, geo-morphological). In a basin-scale hydrological demonstration, the conventional technique for model initiates with getting contour map, DEM, LULC maps, soils maps, and meteorological information. Usually, the specialist's past involvement with these datasets determines if they would be best suited for utilization in a specific scenario^9^. Additional multifaceted complexity is that different scientific networks have varying information sources and control strategies, which makes the interpretation of these data by researchers unfamiliar to the basin physically, exceedingly unreliable. Whilst preparing the LULC with satellite imagery it was noticed that the imagery could be misleading on several occasions. It was noticed that the actual field-collected data for LULC is much more efficient and accurate than satellite imagery. Usually, the researcher’s involvement with a region’s LULC determines the suitability for utilization in a specific scenario. Frequently more updated, or progressively reasonable information items do not exist. In the current scenario familiarity with the actual field conditions helped in creating a fairly accurate recognition of the basin characteristics. It was also noted during the survey that the canals in various places were subject to encroachment, heavy silting, and wastes (plastic, domestic garbage, slaughterhouse waste) that were dumped into the canals which resulted in complete impediment of canal hydraulics in certain regions of the drainage basin.

## Conclusions

A deep insight into the irrigation requirements, water availability, socio-economic statistics, geographical conditions, the agricultural scenario, and meteorological parameters provides a notion that though the water entering the canals as a result of tidal backwater influx might theoretically seem to suffice the irrigation requirements for the winter rice crop, the intensification and high-density production in the region is not prevalent mostly due to the lack of good distribution. An improved canal network would provide a much more increase in the overall productivity of crops in the region. The impact of encroachment, dumping of waste along the lack of maintenance makes the canal network less efficient. Also, control over the tidal backwater returning into the river during the low tide results in a huge loss of water which could be controlled by some minor adjustments and tweaks in the system operation. The model increased the winter cropping great extent and likewise the farmers' income by nearly ten times. Nevertheless, apart from cultivation, the excess water could be a major boost to several other factors like small scale industries and aquaculture, which will greatly improve the socio-economic conditions of an entire region.

The influence of tidal backwater feeding in the non-monsoon period not only allows for multi-cropping and aquaculture but also acts as an active waste and flood water drainage network for the region. The maximization of sustainable use of natural resources is the key to the economic development of any region. The study provides an extensive outlook on the water availability, drainage, and irrigation water supply characteristics of the region but the model can be easily normalized by replicating the model framework whilst changing the specific variable parameters unique to any location to effectively derive results.
